# Understanding COVID-19: the virus

**Published:** 2020-09-01

**Authors:** Jeremy J Hoffman, Adiele E Hoffman

**Affiliations:** 1 Clinical Research Fellow: International Centre for Eye Health, London School of Hygiene & Tropical Medicine, London, UK.; 2 General Practitioner and Distance Learning Tutor: London School of Hygiene & Tropical Medicine, London, UK.

**Novel coronavirus disease 2019 (COVID-19) is the clinical disease caused by SARS-CoV-2, the virus first discovered in Wuhan, China. It has since spread worldwide and by mid-August 2020 had infected over 21.7 million people, resulting in over 770,000 deaths.** ^1^

The virus responsible for COVID-19 is the coronavirus SARS-CoV-2. It was discovered in Wuhan, China and was isolated and identified on 7 January 2020.^1^,^2^ Coronaviruses are single-stranded, spherical RNA viruses, measuring approximately 120 nanometres in diameter – similar in size to influenza virus and HIV, and a little larger than adenoviruses. In coronaviruses, the viral envelope contains three surface proteins: S (spike) protein E (envelope) protein and M (membrane) protein. (Figure 1). It is the projections of the S protein, as seen using electron microscopy (Figure 2), that give coronaviruses their name, as they resemble the solar corona – the halo visible around the sun during a solar eclipse (Figure 3).^2^

The S protein binds to receptors on the surface of the host cell, which enables the virus to fuse with the cell membrane and enter the cell. Once inside the cell, the virus is able to ‘hijack’ the cell so that the host cell produces copies of the virus that then go on to infect other neighbouring cells.^2^

**Figure 1:**
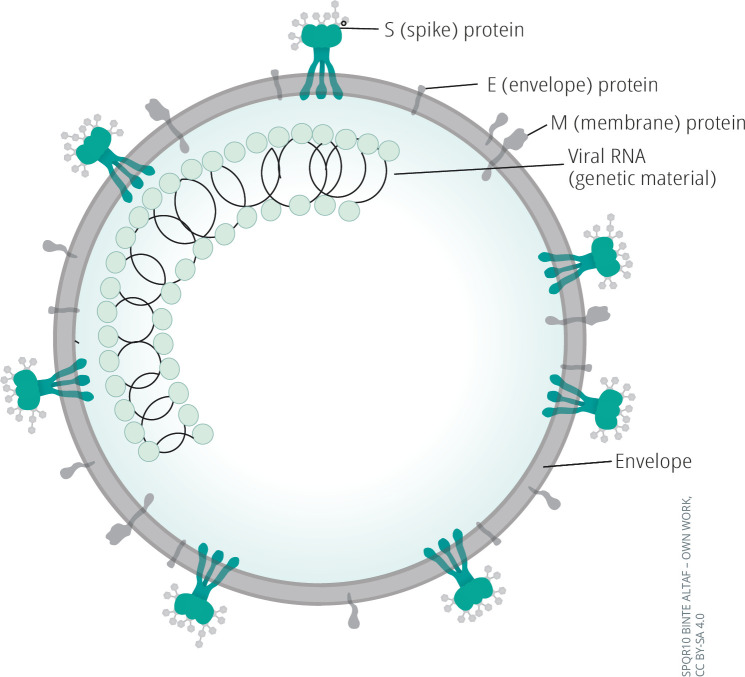
Illustration of SARS-CoV-2 virion

## Transmission

SARS-CoV-2 is primarily transmitted between people via respiratory droplets that are produced when an infected person coughs, sneezes, or speaks. The virus can be transmitted to anyone within a 1 metre radius via their mouth, nose or conjunctiva.

Transmission may also occur via indirect contact, when droplets contaminate surfaces or objects in the immediate environment. Airborne transmission has not been reported under real-life conditions, but may be possible in specific circumstances, such as procedures that generate aerosols (for example, endotracheal intubation).^3–5^

**Figure 2:**
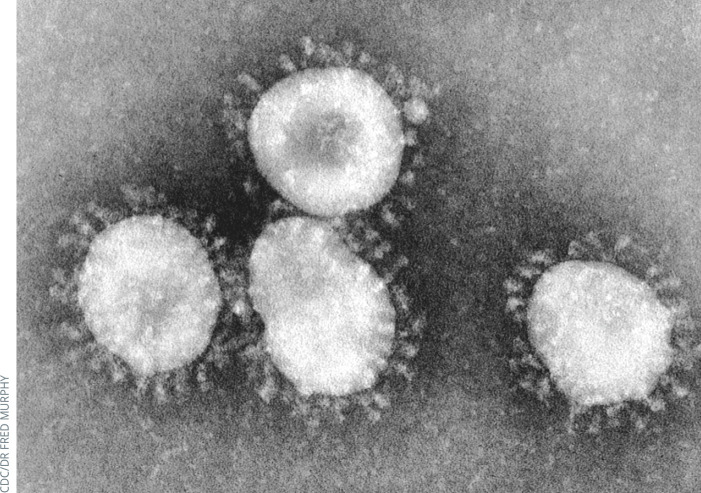
The S (spike) proteins of coronaviruses

**Figure 3:**
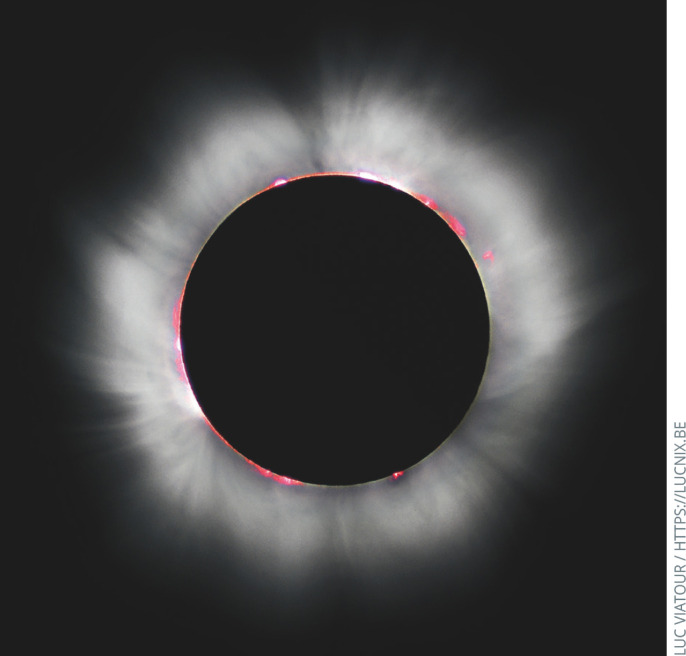
Corona visible during total solar eclipse, as seen in France in 1999

There is evidence that conjunctival secretions and tears from infected patients contain virus RNA and those with conjunctival symptoms may pose higher risk.^6^ Some studies have also shown that SARS-CoV2 may be present in urine or faeces; however, there have been no reports of transmission through faeces or urine.^7^ Asymptomatic transmission has also been reported.^8^

## Clinical disease: signs, symptoms and course

The key symptoms of COVID-19 are as follows:

1. Common symptoms: fever, dry cough, tiredness (malaise), shortness of breath (dyspnoea)
2. Less common symptoms: aches and pains (myalgia), nasal congestion, headache, sore throat, diarrhoea, loss of taste or smell (anosmia)
3. Uncommon symptoms, but described in cases: conjunctivitis, skin rashes, discolouration of fingers or toes.

The incubation period is between 2 and 14 days, with most people developing symptoms on day 5 or day 6.^9^ Fever is the most commonly reported symptom (documented in around 88% of patients), but not always at initial presentation.^10^ A dry cough is present in 66% of patients, although a large minority do have sputum production, and it frequently persists for longer than five days. Gastro-intestinal symptoms and nasal congestion are rarer and have each been reported in about 5% of patients. Difficulty breathing (dyspnoea) is a well-recognised feature, indicating and prognosticating more severe disease. Loss of the sense of smell (anosmia) has also been reported as a strong predictor for COVID-19.^11^ Conjunctivitis has only been reported in 0.8% of cases; this is in keeping with other coronaviruses which are also known to (infrequently) cause conjunctivitis.^12^,^13^

Around 80% of people who become infected will have mild or asymptomatic disease and 14% will have a more severe course, with 5% requiring critical care.^11^ It is worth noting that most of the published studies characterising the symptoms of COVID-19 have been done in the hospital setting and so are likely to have captured a more severe spectrum of disease. Case series documenting milder disease have shown the variability in symptoms from asymptomatic to minor fever or respiratory symptoms, with a highly variable illness duration.^14^,^15^

## Prognostic factors

Although the infectious dose (the number of viral particles needed to establish an infection) for COVID-19 is as yet unknown, it is likely to be low given the rapid spread.^16^ Recent data suggests that higher viral loads isolated in patients is a poor prognostic indicator, which has led to concerns that a high initial infectious dose, to which healthcare workers are likely to be exposed, might lead to higher viral loads and poorer outcomes. There is insufficient evidence to back this up at the moment, although studies have shown this to be the case for influenza A.^17^

Groups that have been shown to be at higher risk of more severe disease include the elderly, particularly those >80 years old, who have a fatality rate of 14.8%, compared to a documented 2.3% total case fatality rate. Other at-risk groups include smokers, men and people with any underlying comorbidity, especially chronic kidney disease, chronic obstructive pulmonary disease and cerebrovascular disease.^18^

**COVID-19 transmission update**

The World Health Organization (WHO) published a new scientific brief on COVID-19 transmission on 9 July. According to the brief, some outbreak reports related to crowded spaces indoors have suggested the possibility of aerosol transmission, combined with droplet transmission; for example, during choir practice, in restaurants, or in fitness classes. The full brief is available at **www.who.int/news-room/commentaries/detail/transmission-of-sars-cov-2-implications-for-infection-prevention-precautions**

## Disease control and prevention

The spread of respiratory viruses can be prevented by hygienic measures such as handwashing, wearing of personal protective equipment (PPE) and by isolating people who are infected. for the 10–14 days it takes for the virus to clear (depending on the severity of the illness.^19^ A Cochrane review article on this subject found that surgical masks or N95 respirators were the most consistent and comprehensive measures to stop the spread of infection.^20^ The subject of face masks has been hotly debated, with many countries now legislating requiring members of the public to wear masks outside the home. In health care settings, WHO recommends frequent hand hygiene, cough etiquette, environmental cleaning, maintaining physical distancing and training health care personnel in the rational and appropriate use of PPE.^3^

## Testing for SARS-CoV-2

Many tests have been developed and granted emergency approval for detecting infection with SARS-CoV-2, with more being developed on a weekly basis.^21^,^22^

Testing can involve:

- Looking for evidence that the virus is **currently active** in the body; this involves looking for viral RNA and is known as **antigen testing**
- Looking for evidence that someone has been **infected previously** and has developed some level of immunity against the virus, e.g., by producing antibodies; this is known **antibody testing**.

An **antigen** is any substance that causes your immune system to produce **antibodies** against it; in this instance the antigen is the SARS-CoV-2 virus.

The tests fall into four main technical categories:

1. Reverse transcription polymerase chain reaction (RT-PCR) (currently the standard detection test for active SARS-CoV-2 infection)
2. Loop-mediated isothermal amplification (LAMP)
3. Lateral flow
4. Enzyme-linked immunosorbent assay (ELISA).

These are outlined in more detail below.

### 1. Reverse Transcription Polymerase Chain Reaction (antigen test)

RT-PCR can be used to detect the virus’s single-stranded RNA genome, which may be present in an individual actively infected with SARS-CoV-2. It is a commonly used diagnostic test that has been in widespread use in research and medicine for over two decades. It is currently the most widely used of the diagnostic tests for SARS-CoV-2. ^21^,^22^

**Figure 4:**
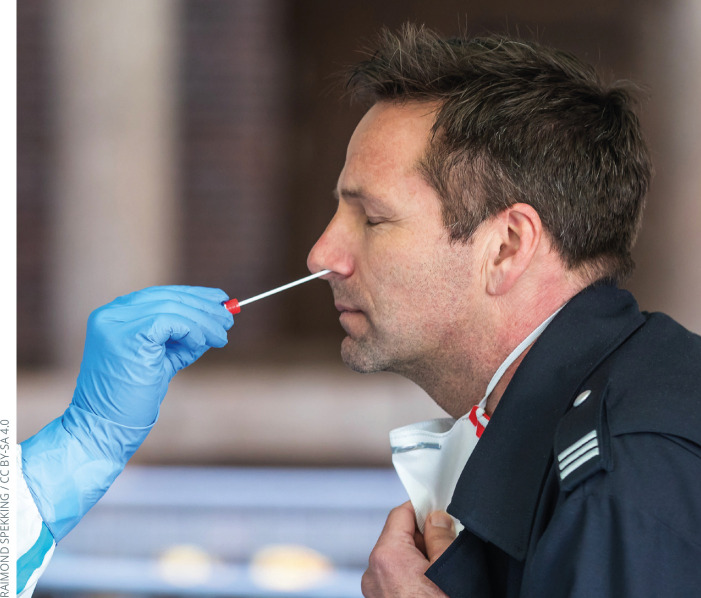
Demonstration of a nasopharyngeal swab for COVID-19 testing being performed in Germany

Practically, this test is performed by taking samples from the nasopharynx, oropharynx and/or sputum and sending these to a designated laboratory (Figure 4). These samples will contain a mixture of the patient’s DNA and any viral RNA that may be present. Additional molecules such as fat, proteins and DNA are destroyed and removed using chemicals. The enzyme in the test, reverse transcriptase, creates a complementary DNA copy of the RNA. Specific regions of the DNA are then isolated with “primers” that have been designed to only detect the complementary DNA of SARS-CoV-2. This is then amplified by using DNA polymerase to synthesise new DNA strands from the deoxynucleoside triphosphates. The PCR machine cycles the test temperature so that, ultimately, 35 billion copies of viral DNA are made for each strand of viral RNA that was initially present. Fluorescent markers then bind to the amplified DNA, emitting light that is read by the PCR machine to give the test result. Once the light intensity is above a defined threshold, the test is deemed positive. It is also possible to get a quantitative estimate of the viral load in the patient’s sample from the number of PCR cycles that were required to give the positive result.

**“Many tests have been developed and granted emergency approval for detecting infection with SARS-CoV-2, with more being developed on a weekly basis.”**

RT-PCR is considered the “gold standard” for detecting SARS-CoV-2. When swabs have been taken appropriately, it is a very sensitive test that can detect current infections of the disease, allowing clinicians to determine who is infected and who is not. However, it relies on active infection and **viral shedding**, so will miss patients who have cleared the infection and recovered, or those who have been recently infected and have not yet started to shed virus. Viral shedding varies in location between individuals and across time, meaning that on one occasion swabs may be positive from sputum but not from the nasopharynx, potentially leading to false negative results.^21^,^22^ According to limited data in the literature, the sensitivity ranges from 56–83%.^23^ With a high prevalence of SARS-CoV-2 in the population, the negative predictive value will decrease. A negative result should therefore be interpreted with caution. Furthermore, it requires expensive equipment and reagents that are not widely available in resource-limited settings, meaning that samples must be sent to a centralised laboratory. This can result in a testing turn-around time of over 48 hours.^21^,^22^

**Viral shedding** takes place when a virus replicates inside the body and is released into the environment.

### 2. Loop-Mediated Isothermal Amplification (LAMP) (antigen test)

LAMP is a newer technology than RT-PCR and uses a similar approach to detecting viral genetic material. However, unlike PCR it performs the testing at a set temperature without cycling. The amount of DNA produced this way is much greater and the result can be visualised without needing a machine to interpret the results, as the reaction mixture turns cloudy due to the production of the chemical magnesium pyrophosphate. Like with PCR, fluorescent dyes can be added to increase the accuracy, so that when a given threshold of light intensity is produced, the test result is deemed positive.^21^,^22^

As LAMP works on the same principle of detecting the viral RNA, it has similar advantages and disadvantages to RT-PCR above in detecting individuals actively infected with the virus. It is also dependent on a swab capturing viral RNA. However, it is cheaper and easier to perform with results visible by eye, meaning it can be performed at hospital laboratories, with results available within 2–3 hours. This reduces the testing turnaround time and may make this test more appropriate to resource-limited settings. However, there is far less evidence relating to the accuracy of these tests and they are still being assessed in clinical settings.

### 3. Lateral flow (antibody test)

These tests are commonly referred to as “antibody tests” or “point-of-care tests” and use the same technology used in urine pregnancy tests and other rapid diagnostic tests. Unlike the two tests described above, they are designed to detect the patient’s antibodies to the virus circulating in the bloodstream, rather than the virus (or viral RNA) present in secretions in the throat or nose.^21^,^22^

A finger-prick of blood is dropped onto a small, absorbent pad in the test device, whilst a small amount of ‘buffer’ solution is added to carry the blood across the device. If there are antibodies against SARS-CoV-2 present in the blood, these bind to specific chemical antigens embedded in lines on the test device. Once captured on these lines, a colour change happens, meaning that the clinician can read off the result. There are usually three such lines: one for IgG antibodies, one for IgM antibodies, and a control line. The control line indicates that the test was performed correctly. Results are available within about 20 minutes.

It usually takes at least 4–5 days for an infected person to produce IgM antibodies. In one study, a total of 90% of infected patients tested positive for IgM antibody tests by days 11–24. IgG antibodies are created later and can be detected several weeks after the initial infection.^21^,^22^ It is not known how long IgG antibodies remain present.^24^ As these tests rely on patients’ antibodies to be present, the test will be negative early in a patient’s illness. If positive, this could be because they are currently infected or because they had the infection in the past. There are also concerns regarding a low specificity, i.e., a relatively large number of people who test positive for antibodies even though they haven’t had COVID-19 (false positives); this potentially gives people a false sense of security that they are immune. Some tests detect antibodies to other coronaviruses that can cause relatively harmless upper respiratory tract infections. Testing kits are currently expensive and labour intensive if many tests are to be performed.

### 4. Enzyme-Linked Immunoabsorbent Assay (ELISA) (antigen and antibody test)

An ELISA is a frequently performed biochemical test that detects either antigens or antibodies of interest. Within the COVID-19 setting, tests have been developed to detect antibodies to SARS-CoV-2, similar to that described in the lateral flow testing above. ELISAs use enzymes that are linked to antibodies that are able to specifically bind to either IgM or IgG anti-SARS-CoV-2 antibodies. When they bind, a colour change occurs, the degree of which can be measured by a machine. ^21^,^22^

As antibody tests, these share the same advantages and disadvantages of the lateral flow testing, allowing people to know whether they have been infected at some point in the past. It has the advantages, over lateral flow, of being cheaper and more easily scaled up to allow larger numbers of patients to be tested. However, these tests are still being developed for SARS-CoV-2 and diagnostic accuracy indices are currently limited.

## Comparison with other diseases

It is important to note that when we talk about ‘the virus’ within the context of COVID-19, we are specifically referring to the SARS-CoV-2 virus. It should not be confused with other viruses that have caused epidemics or pandemics recently.

Although there have been a number of viral epidemics in the 21st century, there has only been one other official pandemic this century: the 2009 H1N1 influenza pandemic. However, it is important to consider the current COVID-19 pandemic within the context of other recent epidemics to place it in context and also see what has been learned from previous experience.

### Severe Acute Respiratory Syndrome (SARS) epidemic (2002–2004)

SARS shares a number of similarities with COVID-19: they are both coronaviruses, both first presented in China, both have likely originated from animals (known as zoonosis), and both spread through respiratory droplets.^25^ However, there are also a number of differences. The cases of SARS were more severe than what is currently being seen in COVID-19, which helped with contact tracing and, eventually, containing the spread of the virus. The mortality rate was also higher, at about 9.6%. However, the virus itself seemed less able to persist in the human population. As a result, there were only 8,098 cases reported across 29 countries. Most transmission occurred within the hospital setting.^25^

**Figure 5:**
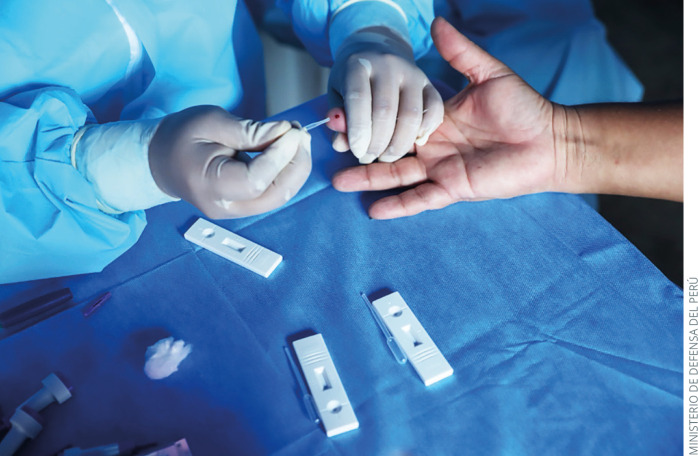
A point-of-care rapid antibody test being performed in Peru. A blood droplet is collected by a pipette

### Middle East Respiratory Syndrome (MERS) epidemic (2012–present)

Like SARS, the MERS epidemic is also a zoonotic coronavirus that causes fever and cough, as well as poor clinical outcomes that are associated with older age and comorbidities. The MERS outbreak originated in Saudi Arabia in 2012 and is still not contained. To date, there have been 2,494 confirmed cases and 858 deaths across 27 countries, with a case fatality rate of 34.4%. Like SARS, most transmission is nosocomial, with limited community transmission – in contrast to COVID-19.^25^

### H1N1 Influenza pandemic (2019)

As the only other pandemic of the twenty-first century, the H1N1 influenza pandemic infected a quarter of the world’s population and led to over 284,000 deaths. It ran from January 2009 until August 2010. Unlike COVID-19, children were most frequently infected, with 47% of children globally aged between 5 and 19 developing symptoms.^26^ This was also one of the groups with the highest mortality rates. Furthermore, antiviral medications existed for severe infections and the development of a vaccine was relatively straightforward, given that influenza vaccination research and manufacture occurs on an annual basis. Vaccine research started in April 2009, and the vaccine was made available by December 2019.

### Ebola epidemics (2014–2016 and 2018–July 2020)

Ebola virus is an extremely deadly virus that can cause mortality rates of up to 50%.^27^ However, it is only spread through bodily fluids such as sweat, blood and vomit, rather than aerosols. This usually occurs in the later stages of the infection when an individual is already unwell and showing symptoms. This is unlike COVID-19, which may be carried asymptomatically. This in part helped in the control of the virus as it made contact tracing relatively more straightforward. There have been two recent epidemics of Ebola: the first between 2014 and 2016 originating in West Africa, and the second starting in 2018 in the Democratic Republic of Congo and still ongoing. In the 2014–16 outbreak, there were 28,616 cases resulting in 11,310 deaths. As of April 2020, there have been 3,461 cases and 2,279 deaths in the current outbreak in DRC.^27^

### 1981 – present: Human Immunodeficiency Virus (HIV) pandemic

HIV is also occasionally referred to as ‘the virus’ and should therefore not be confused with SARS-CoV-2. The HIV virus, clinical disease and pandemic are very different from the other outbreaks listed above. HIV is a retrovirus that infects CD4 immune cells, leading to immunodeficiency and ultimately Acquired Immunodeficiency Syndrome (AIDS) if not treated. Transmission is through contaminated bodily fluids, meaning it can spread through sexual contact, contaminated needles or blood products. The first cases were recognised in 1981 and since then it has spread globally, with a significant burden of the disease being borne in Africa.^28^ At present, there are over 37.9 million people living with HIV globally, of whom 62% have access to treatment in the form of antiretroviral therapy.^29^ The outbreak has spanned four decades and although there has been significant progress in terms of treatment and prevention, there is still no commercially available vaccine.

## Vaccines and therapies

No vaccine or specific treatment has so far been licenced for COVID-19, although research into these is happening at an unprecedented rate. Regulators are permitting fast-tracked human trials into new possible therapies and vaccines without the need for going through the usual preliminary research and development. Details of these therapies are beyond the scope of this article, but a summary of current research can be found at the Centre for Evidence-Based Medicine.^30^

Human trials of a vaccine have recently commenced, although it is unlikely to be ready for distribution until the end of 2020, at the earliest. A wide number of drugs have been trialled in patients, but case series are small and evidence for their routine use is lacking. Treatment of COVID-19 remains supportive, with severe cases needing supplementary oxygen or non-invasive ventilation. In the most severe patients, admission to an intensive care unit is required, with sedation, intubation and ventilation. A recent clinical trial has demonstrated that dexamethasone can reduce the mortality in severe patients who are ventilated or receiving supplementary oxygen.^31^ Readers are recommended to follow WHO clinical guidance on its use.
